# Role of STAT3 in *In Vitro* Transformation Triggered by TRK Oncogenes

**DOI:** 10.1371/journal.pone.0009446

**Published:** 2010-03-03

**Authors:** Claudia Miranda, Tiziana Fumagalli, Maria Chiara Anania, Maria Grazia Vizioli, Sonia Pagliardini, Marco A. Pierotti, Angela Greco

**Affiliations:** 1 Operative Unit “Molecular Mechanisms”, Department of Experimental Oncology and Molecular Medicine, IRCCS Foundation, Istituto Nazionale dei Tumori, Milan, Italy; 2 Scientific Directorate, IRCCS Foundation, Istituto Nazionale dei Tumori, Milan, Italy; New Mexico State University, United States of America

## Abstract

TRK oncoproteins are chimeric versions of the NTRK1/NGF receptor and display constitutive tyrosine kinase activity leading to transformation of NIH3T3 cells and neuronal differentiation of PC12 cells. Signal Transducer and Activator of Transcription (STAT) 3 is activated in response to cytokines and growth factors and it has been recently identified as a novel signal transducer for TrkA, mediating the functions of NGF in nervous system. In this paper we have investigated STAT3 involvement in signalling induced by TRK oncogenes. We showed that TRK oncogenes trigger STAT3 phosphorylation both on Y705 and S727 residues and STAT3 transcriptional activity. MAPK pathway was involved in the induction of STAT3 phosphorylation. Interestingly, we have shown reduced STAT3 protein level in NIH3T3 transformed foci expressing TRK oncogenes. Overall, we have unveiled a dual role for STAT3 in TRK oncogenes-induced NIH3T3 transformation: i) decreased STAT3 protein levels, driven by TRK oncoproteins activity, are associated to morphological transformation; ii) residual STAT3 transcriptional activity is required for cell growth.

## Introduction

TRK oncogenes, isolated from papillary thyroid tumors [1], represent rearranged versions of the *NTRK1* gene, coding for Nerve Growth Factor (NGF) receptor, also named TRKA. Chromosomal rearrangements juxtapose the NTRK1 tyrosine kinase domain to N-terminal portion of unrelated genes, generating chimeric oncogenes named TRK, TRK-T1, TRK-T2 and TRK-T3 [2]. TRK oncoproteins display constitutive tyrosine kinase activity, promoted by coiled-coil domains provided by the activating genes [2], thus mimicking the biochemical and biological effects of NTRK1 receptor activated by NGF [3], [4]. The mechanism of action of TRK oncoproteins and their intracellular signal transduction has been in part elucidated. This has been performed by expressing the TRK oncogenes in different mammalian cell systems; among these, NIH3T3 and PC12 cell lines, which represent a useful model for studying *in vitro* oncogene-triggered transformation and neuronal-like differentiation, respectively [3], [4]. Interactions of TRK oncoproteins with several signal transducers (SHC, FRS2, FRS3, IRS1, IRS2, PLC-γ and SHP-1), leading to activation of the MAPK (Mitogen-Activated Protein Kinase) pathway, have been identified, and their role in the process of oncogenic transformation has been elucidated [5]–[8]. Nevertheless, more studies are required in order to fully dissect the mechanism of action of TRK oncogenes.

Signal Transducer and Activator of Transcription (STAT) 3 is a member of a gene transcription protein family initially discovered as cytokine signal transducers, and subsequently found involved in the signaling of growth factor tyrosine kinase receptors, cytoplasmic tyrosine kinases and oncogenes [9]–[11]. Conventional STAT3 activation consists in phosphorylation on a single tyrosine residue (Y705) resulting in dimerization of the STAT1/3 transcription factors, translocation into the nucleus and transcriptional activation of target genes [10], [12]. In addition to phosphorylation on Y705, phosphorylation on serine 727 (S727) residue enhances the transcriptional activity of STAT3. Cooperation of phosphorylated Y705 and S727 is necessary for full activation of STAT3 [13]; on the other hand, S727 phosphorylation is sufficient to activate STAT3 signaling independently of Y705 phosphorylation [14]–[16]. Different cellular systems may affect STAT3 Y705 or S727 phosphorylation as different protein kinases may be implicated [17], [18].

STAT3 is involved in a variety of biological processes including cell proliferation and carcinogenesis [19]. Constitutive STAT3 activation has been found in multiple types of tumors, including melanoma, prostate cancer, head and neck squamous cell carcinoma [20], [21], and inactivation of STAT3 has been found to inhibit a variety of malignancies [19], [22]. STAT3 is involved in the signal transduction pathway of several oncogenic tyrosine kinases, including v-src, NPM-ALK, RET-MEN2A and RET/PTC oncogenes [11], [23]–[25].

In addition to its nuclear function as transcription factor, STAT3 exerts transcriptional independent function in regulating cell migration [26]. In particular, cytoplasmic STAT3 plays a role in mediating microtubules (MTs) dynamics through functional interaction with stathmin, a key MT-destabilizing protein [27], [28].

Recent studies identified STAT3 as a novel signal transducer for TrkA, suggesting a role of STAT3 in the nervous system [29]. In PC12 cells, activation of TrkA by NGF triggered STAT3 phosphorylation at S727, enhancing its DNA binding and transcriptional activities; by contrast, no STAT3 Y705 phosphorylation was induced by NGF treatment. STAT3 activation induced by TrkA mediates several downstream functions of NGF signaling, in particular cyclin D1 expression and neuronal differentiation.

In this paper we investigated STAT3 involvement in the signalling triggered by TRK oncogenes in different cellular systems, such as PC12 and NIH3T3 cells. In particular we showed that TRK oncogenes induced Stat3 phosphorylation, both on Y705 and S727, and Stat3 transcriptional activity, partially through MAPK pathway. We have unveiled a dual role for STAT3 in TRK oncogenes-induced NIH3T3 transformation: i) decreased Stat3 protein levels, driven by TRK oncoproteins activity, are associated to morphological transformation; ii) residual Stat3 transcriptional activity is required for cell growth.

## Results

### TRK Oncogenes Induce STAT3 Phoshorylation and Transcriptional Activity

A recent report has shown that Stat3 is involved in the intracellular signalling triggered by the TrkA receptor. In PC12 cells the stimulation of TrkA by NGF induces Stat3 phosphorylation on S727 but not on Y705, enhances its DNA binding and transcriptional activities; therefore Stat3 mediates several downstream effects of NGF, including neuronal differentiation [29].

**Figure 1:**
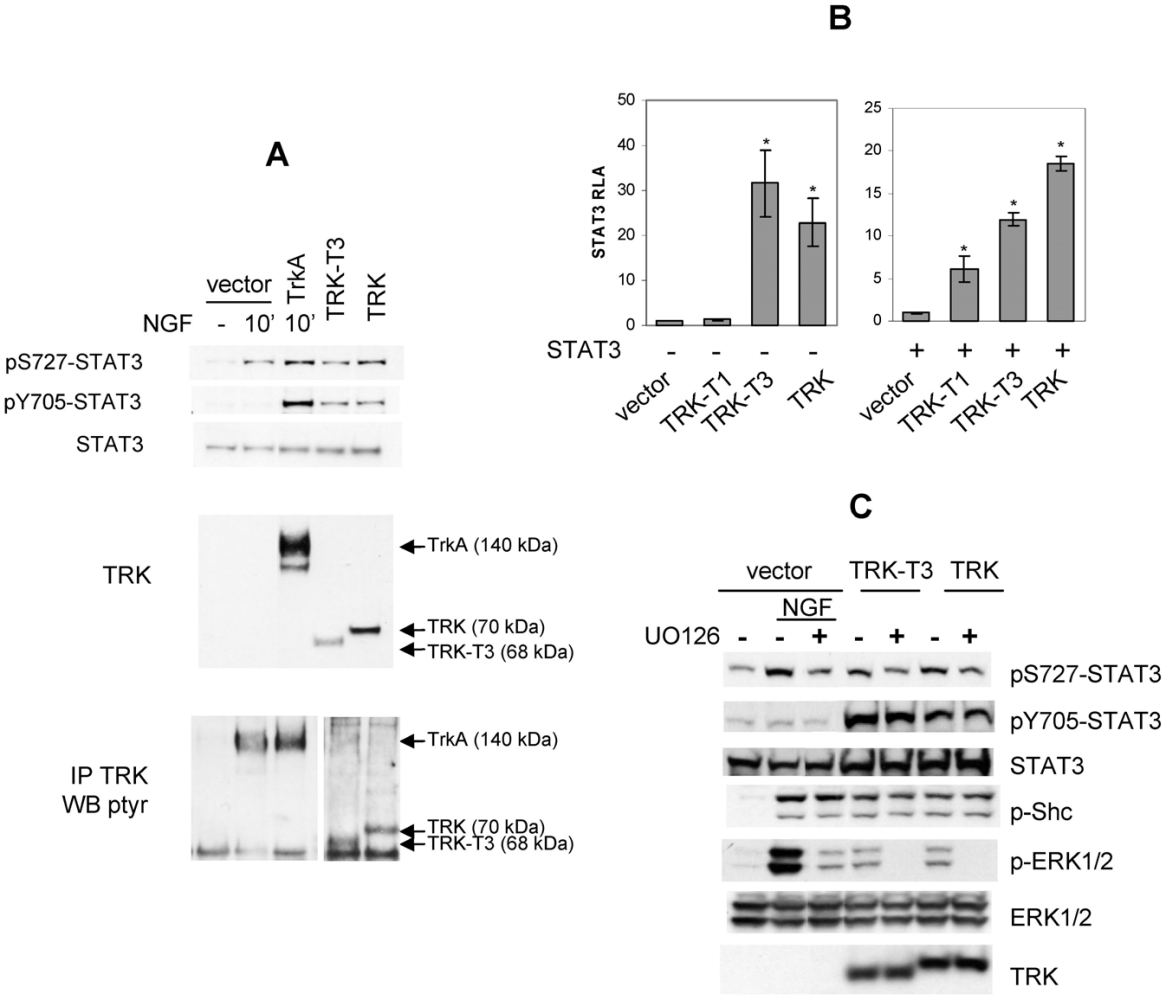
STAT3 activation by TRK oncogenes. (**A**) Western blot analysis of PC12 cells transfected with empty pRC/CMV vector, TRKA, TRK-T3, and TRK cDNAs. NGF treatment (50ng/ml, 10′) is indicated. Cell lysates and immunocomplexes were separated by SDS PAGE as described in Material and Methods and immunoblotted with indicated antibodies. In the bottom panel two distinct exposures of the same blot were used for documenting TRKA or TRK oncoproteins phosphorylation. (**B**) HeLa cells were co-transfected with pM67 and pRL-TK in combination with the indicated TRK oncogene cDNAs in the absence (left graph) or presence (right graph) of STAT3 cDNA and assayed for STAT3-dependent luciferase activity 48 hours later. Activity is expressed as the ratio of luciferase/renilla activity, and reported as fold-inductions over empty vector (left panel) or STAT3 (right panel) (RLA: Relative Luciferase Activity). The data represent the mean values ± SD of triplicate samples. Similar results were obtained in three independent experiments. (**C**) MAPK involvement in TRK-induced Stat3 phosphorylation. Western blot analysis of PC12 cells transfected with empty pRC/CMV vector, TRK-T3 or TRK cDNAs. NGF (10′, 50ng/ml) and UO126 (16 hr, 10 µM) treatments are indicated. Immunoblotting was performed with the indicated antibodies.

We have previously shown that TRK oncogenes, chimeric versions of the TRKA receptor containing its tyrosine kinase domain [30], induce neuronal like differentiation of PC12 cells thus mimicking the effect of NGF treatment [3]. To investigate whether STAT3 is involved in signalling of TRK oncogenes, we analyzed Stat3 phosphorylation status by Western blot analysis with anti-phosphoSTAT3 S727 and Y705 antibodies in PC12 cells transiently transfected with TRK oncogenes (Figure 1A). In mock transfected PC12 cells NGF stimulation of endogenous TrkA receptor induced Stat3 phosphorylation on S727 but not on Y705 residues, in accordance to literature data [29]. By contrast, the expression of TRK and TRK-T3 oncogenes induced Stat3 phosphorylation on both S727 and Y705 residues. This discrepancy is related to protein expression level; in fact phosphorylation of both Stat3 residues was observed in NGF-treated PC12 cells overexpressing the TRKA receptor. Expression levels of transfected TRKA receptor and TRK oncoproteins are shown in the second panel; in these conditions endogenous TrkA expression was not detectable, however its tyrosine phosphorylation was observed in TRK immunocomplexes following NGF stimulation (Figure 1A bottom panel).

STAT3 phosphorylation promotes its dimerization and nuclear translocation, followed by transcription of target genes [10], [12]. To analyze whether TRK oncogenes-induced STAT3 phosphorylation correlated with STAT3 dependent transcriptional activity, we performed STAT3 transactivation luciferase assay. HeLa cells were transfected with pM67 plasmid, containing the luciferase gene under the control of STAT3 responsive elements, in combination with TRK constructs or empty vector, and assessed for STAT3-dependent transactivation. In the luciferase assay shown in Figure 1B (left panel), a marked effect of TRK-T3 and TRK oncogenes was observed, as they induced a 30 and 22 fold increase of STAT3 transcriptional activity relative to control, whereas TRK-T1 effect was undetectable. Overexpression of STAT3 by cDNA transfection (Figure 1B, right panel) unveiled that also TRK-T1 is able to induce STAT3 transcriptional activity, although with reduced efficiency with respect to TRK-T3 and TRK oncogenes.

In PC12 cells NGF-induced Stat3 S727 phosphorylation is mediated by distinct signaling pathways, including MAPK [29]. As MAPK pathway is involved in TRK oncoproteins signalling, we evaluated the effect of its inhibition on TRK oncogenes-induced Stat3 phosphorylation in PC12 cells (Figure 1C). In mock transfected PC12 cells, treatment with the MEK inhibitor UO126 reduced Stat3 S727 phosphorylation induced by NGF stimulation of endogenous TrkA receptor, in accordance to literature data [29]. In PC12 cells expressing TRK and TRK-T3 oncogenes, UO126 treatment reduced Stat3 phosphorylation on S727; by contrast, no effect was observed on Stat3 Y705 phosphorylation. TrkA, TRK-T3 and TRK kinase activity was not affected by the MEK inhibitor, as demonstrated by their capability to phosphorylate Shc adaptor. Phosphorylated and total ERK1/2, and TRK oncoproteins levels are shown in lower panels as control. On the whole data reported in Figure 1C suggest that in PC12 cells ERKs activation, induced by stimulated TrkA receptor and TRK oncogenes, is responsible for STAT3 S727 but not Y705 phosphorylation.

### Analysis of Stat3 in NIH3T3 Cells Expressing TRK Oncoproteins

**Figure 2:**
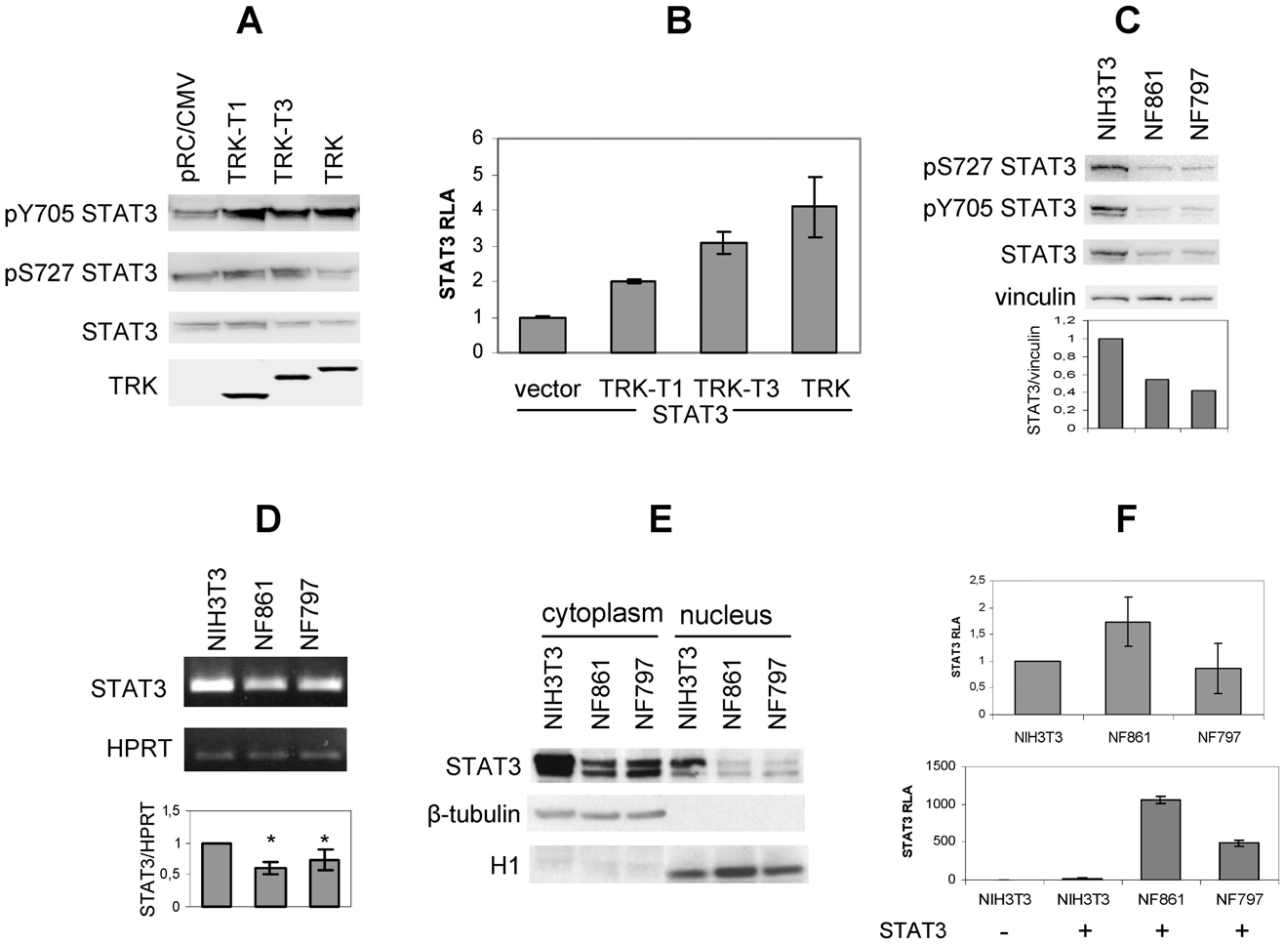
Analysis of Stat3 in NIH3T3 cells expressing TRK oncoproteins. (**A**) Western blot analysis of NIH3T3 cells transiently transfected with TRK oncogenes. Cells were transfected as described in Materials and Methods, cell lysates and immunocomplexes separated by SDS PAGE and immunoblotted with the indicated antibodies. (**B**) NIH3T3 cells were co-transfected with pM67 and pRL-TK in combination with the indicated TRK oncogene and STAT3 constructs and assayed for STAT3-dependent luciferase activity 48 hours later. Activity is expressed as the ratio of luciferase/renilla activity, and reported as fold-inductions over STAT3 basal activity (RLA: Relative Luciferase Activity). The data represent the mean values ± SD of triplicate samples. Similar results were obtained in two independent experiments. (**C**) Cell extracts from NIH3T3, NF861 and NF797 cells were immunoblotted with the indicated antibodies. Images were acquired by Biorad ChemiDoc and densitometric analysis of the bands was performed by Image Quant software. Data are expressed as ratio STAT3/vinculin and normalized for NIH3T3 sample. (**D**) RT PCR analysis of Stat3 expression. Stat3 and HPRT fragments were obtained as described in Material and methods and separated on 2% agarose gel. Images were acquired by Biorad GelDoc and densitometric analysis of the bands was performed by Image Quant software. Densitometric values are expressed as ratio Stat3/HPRT and normalized for NIH3T3 sample. Data represent the mean values ± SD of four independent experiments. (**E**) Cytoplasmic and nuclear extracts from NIH3T3, NF861 and NF797 cells were obtained as described in Material and methods and subjected to Western blot analysis with the indicated antibodies. (**F**) NIH3T3, NF861 and NF797 cells were co-transfected with pM67 and pRL-TK in combination with the indicated TRK oncogene, alone (upper panel) or in combination with STAT3 construct (bottom panel) and assayed for STAT3-dependent luciferase activity 48 hours later. Activity is expressed as the ratio of luciferase/renilla activity, and reported as fold-inductions over STAT3 activity in NIH3T3 cells. Experiments were performed in triplicates; data represent the mean values of three independent experiments. RLA: Relative Luciferase Activity.

To investigate the role of STAT3 in TRK oncogenes-induced *in vitro* transformation, we analysed Stat3 phosphorylation in NIH3T3 cells expressing TRK oncoproteins. As shown in Figure 2A, transient expression of TRK-T1, TRK-T3 and TRK oncogenes increased Stat3 phosphorylation on Y705 but not on S727. The luciferase reporter assay in Figure 2B showed that STAT3-dependent transcriptional activity was increased in cells expressing TRK oncogenes, indicating their capability to activate STAT3.

We next analyzed Stat3 status in NF861 and NF797 cells, NIH3T3-derived transformed foci stably expressing TRK-T1 and TRK-T3 oncogenes respectively [4], [31] (Figure 2C). Surprisingly, by Western blot analysis we observed that Stat3 protein level was reduced in NF861 and NF797 compared to NIH3T3 by approximately 50%, as indicated by densitometric analysis of Stat3 protein normalized for vinculin levels. The signals detected with anti phosphoSTAT3 antibodies correlate to Stat3 protein amounts. Vinculin levels, shown as control, indicated similar protein amount load.

To explore the mechanism involved in Stat3 down-regulation in NIH3T3-derived foci, we investigated Stat3 mRNA expression by RT-PCR in NIH3T3, NF861 and NF797 cells. Densitometric analysis of Stat3 PCR products, normalized for HPRT levels, showed a reduction of STAT3 mRNA by approximately 50% in NF861 and NF797 compared to NIH3T3 cells (Figure 2D), thus suggesting a down-regulation of Stat3 at the transcriptional level.

Following activation by phosphorylation, STAT3 translocates to the nucleus where it acts as a transcription factor; therefore nuclear levels of STAT3 are important for its transcriptional activity. To investigate nuclear Stat3 levels in parental NIH3T3 and NF861 and NF797 transformed cells, we performed cell fractionation and assayed for STAT3 protein levels. As shown in Figure 2E, reduced levels of Stat3 protein were confirmed in both cytoplasm and nucleus of NF861 and NF797 with respect to NIH3T3 cells. Comparison of cytoplasmic versus nuclear Stat3 levels did not highlight significant differences among cell lines in the distribution of Stat3 protein in the two fractions. α-tubulin and histone H1 levels are shown as controls for cytoplasmic and nuclear fractions, respectively.

To assess whether different Stat3 protein amounts result in different Stat3 transcriptional activity in NIH3T3 parental and transformed cell lines, we performed STAT3-dependent luciferase reporter assay. NIH3T3, NF861 and NF797 cells were transfected with pM67 luciferase reporter vector and assessed for STAT3-dependent transactivation. As shown in Figure 2F (top), despite the reduced Stat3 protein levels (see Figure 2C) the Stat3 activity in NF861 and NF797 foci is equivalent to that in NIH3T3 cells. In addition, overexpression of STAT3 produced a drastic increase of luciferase activity in NF861 and NF797 compared to NIH3T3 cells (Figure 2F, bottom).

### Reduction of Stat3 Level Is Associated with Morphological Transformation Induced by TRK Oncogenes Activity

**Figure 3:**
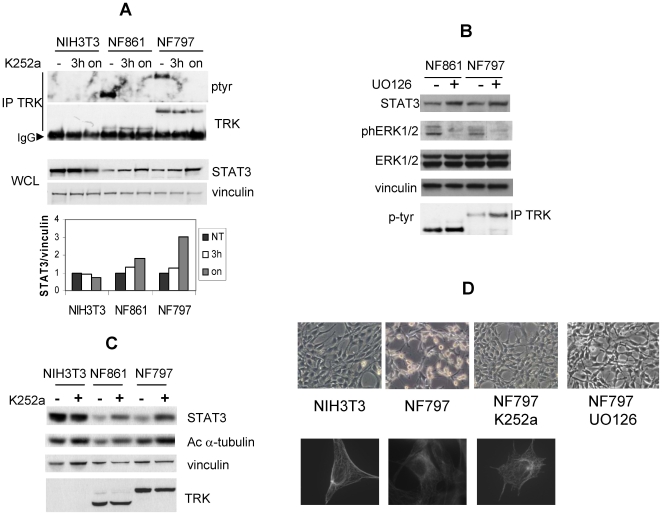
Reduction of Stat3 level is associated to morphological transformation induced by TRK oncogenes activity. (**A**) NIH3T3, NF861 and NF797 cells were treated with K252a (200nM) for the indicated time; cell lysates were processed and immunoblotted with the indicated antibodies. Densitometric analysis of the bands is reported in the bottom panel. Images were acquired by Biorad ChemiDoc and analysed with Image Quant software. Data are reported as ratio of STAT3/vinculin and normalized over untreated samples for any cell line. (**B**) Cell extracts from NIH3T3, NF861 and NF797 cells treated with K252a (200nM, 16 hr) were analyzed by Western blot with the indicated antibodies. (**C**) Cell extracts from NIH3T3, NF861 and NF797 cells treated with UO126 (10 µM, 16 hr) were analyzed by Western blot with the indicated antibodies. (**D**) Phase contrast images (top) and α-tubulin immunostaining (bottom) of NIH3T3 cells, NF797 cells treated or not with K252a (200nM, overnight) or UO126 (10 µM).

To investigate whether TRK kinase activity accounted for reduced Stat3 levels in NIH3T3 foci expressing TRK oncogenes, we treated NIH3T3, NF861 and NF797 cells with TRK kinase inhibitor K252a, and analysed Stat3 protein levels by Western blotting and densitometry (Figure 3A). Treatment of NF861 and NF797 cells with K252a abrogated TRK oncoproteins tyrosine phosphorylation, as previously reported [6]. No effect of K252a on Stat3 protein level was detected in NIH3T3 cells. On the contrary, K252a increased Stat3 levels in NF861 and NF797 foci. Cells exposed overnight to K252a showed a marked increase of Stat3 level (50% and 70%, respectively) and phenotypic reversion of transformed morphology (Figure 3D), suggesting a correlation between TRK oncoproteins activity, transformed phenotype and low Stat3 levels.

Involvement of MAPK pathway in the regulation of Stat3 protein levels was also investigated (Figure 3B). Inhibition of MAPK pathway by UO126, which cause a partial reversion of the transformed phenotype (Figure 3D), induced an increase of Stat3 protein levels in NF861 and NF797 cells, indicating that MAPK activity triggered by TRK oncogenes is involved in the regulation of Stat3 protein levels. No effect of UO126 treatment was observed on phosphorylated TRK; phosphorylated ERK1/2, total ERK1/2 and vinculin levels are shown in lower panels as controls.

Recently, a role for cytoplasmic STAT3 has been described [26]. In particular, STAT3 has been related to microtubules (MTs) stabilization, by preventing the disassembly of MTs induced by the interacting partner stathmin. To explore a possible correlation between low levels of Stat3 in NF861 and NF797 cells and MTs destabilization, we analyzed the amount of acetylated α-tubulin, a marker of MTs stabilization, in cells untreated or treated with K252a. As shown in Figure 3C, acetylated α-tubulin levels were less abundant in NF861 and NF797 cells compared to NIH3T3 cells, indicating MTs disorganization in transformed cells. Such disorganization was visible in α-tubulin immunostaining of NF797 cells and is in keeping with their transformed phenotype (Figure 3D). Following overnight treatment with K252a, the fraction of acetylated α-tubulin raised in NF861 and NF797, along with Stat3 protein increase (Figure 3C). Concomitantly, NF861 and NF797 cells display MTs organization and cell morphology similar to naïve NIH3T3 cells (Figure 3D). This suggests that cell shape, MTs stabilization and Stat3 levels may be linked in NIH3T3-derived foci expressing TRK oncogenes.

### Role of Stat3 in TRK-Induced Cell Growth

**Figure 4:**
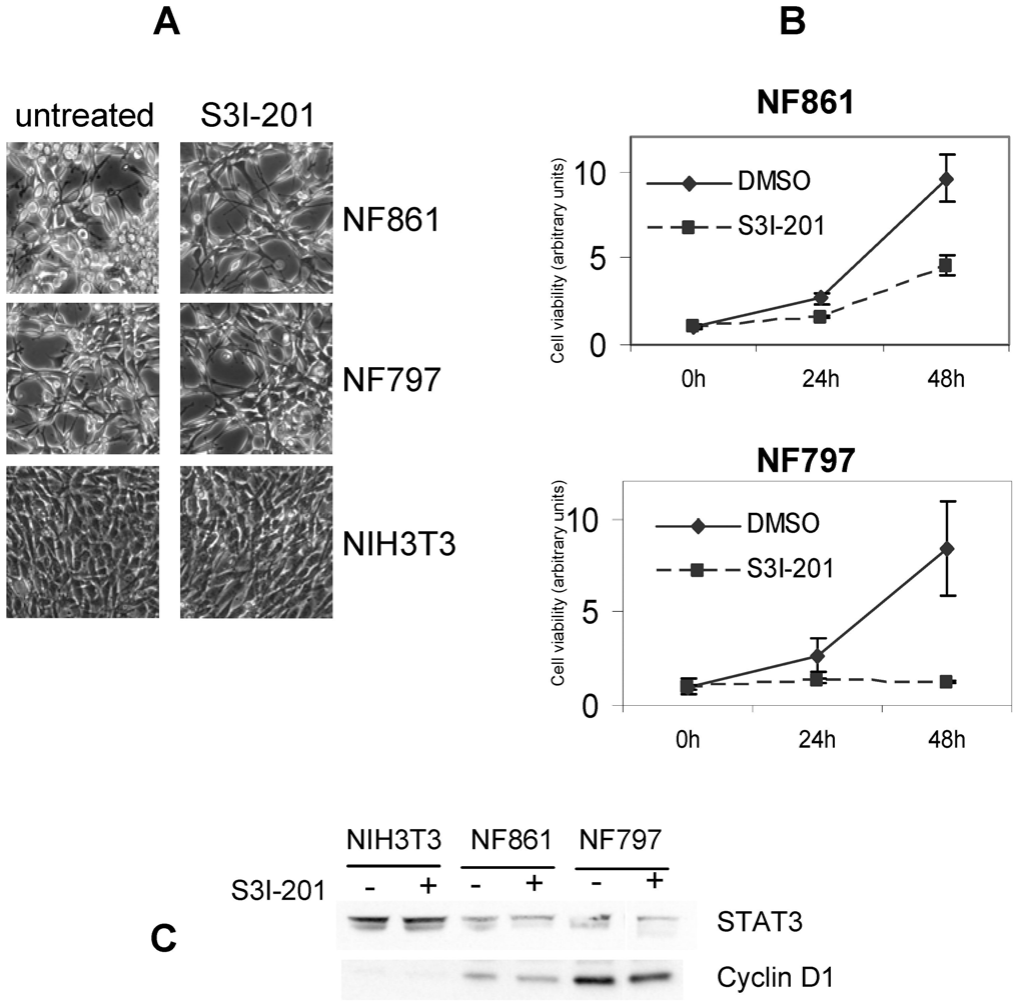
Role of Stat3 in TRK-induced cell growth of NIH3T3 derived foci. (**A**) Phase contrast images of NF861, NF797 and NIH3T3 cells treated or not with S3I-201 (100 µM, 48 hr). (**B**) Cell viability of NF861 and NF797 cells treated with DMSO (0.3%) or S3I-201 (100 µM) determined by the Alamar Blue cell viability assay. (**C**) Western blot analysis of NIH3T3, NF861 and NF797 cells treated with S3I-201 (100 µM, 48 hr). Samples were immunoblotted with the indicated antibodies.

STAT3 protein is activated in response to growth factor and cytokines and promotes cell proliferation and survival [10]. We investigated the effect of inhibition of Stat3 activity in NF861 and NF797 by using S3I-201 (also named NSC 74859), a chemical inhibitor which interferes with STAT3 complex formation and affects its DNA-binding and transcriptional activity [32]. NF861 and NF797 foci, and NIH3T3 cells as control, treated with SI3-201 inhibitor for 48 hours did not show any morphological change (Figure 4A), suggesting that Stat3 transcriptional activity is not involved in foci transformed phenotype. We next determined the effect of S3I-201 an NF861 and NF797 cell growth by fluorometric cell viability assay. As shown in Figure 4B, S3I-201 reduced the growth rate of NF861 and NF797 (40 and 50% reduction at 24 hours; 50 and 85% reduction at 48 hours, respectively), indicating an important role of Stat3 in TRK oncogenes induced cell proliferation. Reduction in cell number after 48 hours treatment with SI3-203 was confirmed by cell count (data not shown). In the same conditions, no evidence of apoptosis or necrosis was observed in NF861 and NF797 cells by FACS analysis (data not shown).

Since cyclin D1 is one of STAT3 major targets and is involved in cell proliferation, we analyzed whether treatment with STAT3 inhibitor may affect cyclin D1 expression in NF861 and NF797 foci compared to NIH3T3 cells. As shown in Figure 4C, cyclin D1 expression was higher in transformed foci than in parental NIH3T3 cells. Treatment with S3I-201 did not cause reduction of cyclin D1 levels in NF861 and NF797, suggesting that cyclin D1 induction by TRK oncogenes may be mainly driven by pathways different from STAT3.

## Discussion

STAT3 is a pleiotropic factor activated by many different signals including cytokines, growth factors and oncogenes, and it mediates cellular proliferation, differentiation, survival and immune function [12]. Recently it has been shown that Stat3 is activated in response to NGF through phosphorylation of S727 residue and mediates its neurotrophic effects [29]. In this work, we have investigated Stat3 involvement in TRK oncogenes signalling in different cellular systems such as PC12 and NIH3T3 cells.

We have observed that in PC12 cells TRK oncogenes expression induced Stat3 phosphorylation on both Y705 and S727 residues, whereas NGF stimulation of endogenous TrkA receptor increased only S727 phosphorylation, in agreement with literature data [29]. This discrepancy is probably due to different expression levels, since overexpression of TRKA receptor in PC12 cells drives also Stat3 phosphorylation on Y705 in response to NGF. In a previous work Ng *et al*, who firstly showed the absence of Y705 Stat3 phosphorylation in NGF stimulated PC12 cells, have hypothesized either the presence of a potential intrinsic dephosphorylation mechanism of Y705, or inhibition of Y705 phosphorylation triggered by phosphorylated S727 [29]. Our findings that Stat3 Y705 phosphorylation is triggered by TRKA receptor and TRK oncoproteins, suggests a dephosphorylation mechanism as the most likely. In this view, overexpression of TRK proteins might perturb the equilibrium of a putative dephosphorylation mechanism which may lead to detection of Y705 phosphorylation. No evidence of direct association between Stat3 and TrkA receptor has been previously shown; similarly, in our experimental conditions, we failed to detect interaction between Stat3 and TRK oncoproteins. Nevertheless, we can not exclude a possible direct phosphorylation of Stat3 on Y705 by TRKA receptor and TRK oncoproteins. More studies will be required to unveil the precise mechanism and the kinase(s) involved in Y705 STAT3 phosphorylation and the pathways implicated. We have determined that in PC12 cells Stat3 activation by TRK oncoproteins requires signalling through MAPK, as UO126 treatment affected Stat3 phosphorylation on S727 but not on Y705. This is in keeping with the hypothesis that Y705 could be phosphorylated either directly by TRK oncoproteins or by alternative pathways.

It has been proposed that phosphorylation of S727 may be mediated by different mechanisms in different experimental cellular systems [13]–[18]. In keeping with this, we have observed that Stat3 S727 is phosphorylated in PC12 but not in NIH3T3 cells transiently transfected with TRK oncogenes.

In a model of *in vitro* transformation represented by NIH3T3 cells transformed by TRK oncogenes (NF861 and NF797) we observed reduction of Stat3 protein levels, but no difference in Stat3 cellular localization in comparison to parental NIH3T3 cells. Reduction of Stat3 in NIH3T3 foci is related to increased microtubules destabilization, as indicated by reduced acetylated α-tubulin and transformed cell morphology. When TRK oncoproteins activity was blocked by K252a inhibitor, NIH3T3 foci underwent morphological reversion and STAT3 protein raised to a level comparable to NIH3T3 cells, indicating that Stat3 reduction in transformed foci correlated to morphological features of transformation driven by TRK kinase activity. We have previously shown that ERK activation is crucial for TRK oncogenic activity: abrogation of signalling through SHC adapter, affecting MAPK activation, impairs TRK-T3 transforming activity [5] and inhibition of MEK by UO126 treatment lead to morphological reversion of NF861 and NF797. Similarly to inhibition of TRK kinase activity, inhibition of MAPK pathway lead to reduction of STAT3 protein level. Interestingly, reduction of STAT3 levels may be common feature of NIH3T3 transformed foci, as it has already been observed in NIH3T3 cells transformed by Ha-rasV12 oncogene [33], and by other oncogenes such as TPR-MET, RET/PTC1 and COLA1/PDGF (our unpublished results).

Our data support the notion that decrease of STAT3 is part of the *in vitro* transformation program as it is associated to the destabilization of MTs typical of transformed NIH3T3 cells. This is in agreement with a recently described transcriptional independent role of STAT3 in mediating MTs dynamics and cell migration through a direct functional interaction with stathmin [27]. An intriguing hypothesis would be that reduced Stat3 levels in the cytoplasm may render stathmin, no longer sequestered by Stat3, more available to interact with tubulin and therefore destabilize MTs. More recently it has been shown that Stat3 modulates Rac1 independently from Stat3 transcriptional activity. Loss of Stat3 expression in mouse embryonic fibroblasts (MEF) drives increased Rac1 activity, and this promotes a random mode of migration by reducing directional persistence and formation of actin stress fibers [34]. It is interesting to note that WT MEF exhibit a classical polarized phenotype, whereas MEFs not expressing Stat3 display a non-polarized phenotype very similar to the one of NIH3T3-derived transformed foci.

In Ha-rasV12 transformed NIH3T3 cells, Stat3 reduction is due to regulation of proteasomal degradation of the protein [33]. This mechanism is not involved in TRK oncogenes-induced Stat3 reduction, as treatment of NF861 and NF797 cells with the proteasome inhibitor lactacystin did not induce any Stat3 protein change (data not shown). Instead, we have observed a reduction of Stat3 mRNA in foci expressing TRK oncogenes compared to NIH3T3 cells, indicating a transcriptional regulation of Stat3 by TRK-T1 and TRK-T3 oncogenes in transformed foci. Several observations [35]–[37] lead to the hypothesis that cyclin D1 overexpression may down-regulate STAT3 expression. This may occur in NIH3T3 foci expressing TRK oncogenes in which cyclin D1 is overexpressed compared to parental NIH3T3 cells.

Despite the reduced protein level, in NF797 and NF861 cells Stat3 transcriptional activity is comparable to parental NIH3T3 cells and it is required for cell growth, as demonstrated by treatment with S3I-201, a compound inhibiting STAT3 transcriptional activity by interfering with dimer formation.

On the whole, the data presented in this work unveil a dual role of Stat3 in *in vitro* transformation triggered by TRK oncogenes: 1) reduced Stat3 levels are linked to morphological transformation typical of NIH3T3 foci; 2) the transcriptional activity of the residual Stat3 amount is necessary for cell growth.

## Materials and Methods

### Plasmids

T3/WT, pC24B (TRK-T1 construct), pDM16 (TRK construct) have been previously described [3]–[5], [38]. pRC-STAT3 flag plasmid [11] was kindly provided by Dr. Darnell (Rockfeller University, NY). For luciferase assay pTATA(TK)Luc4xM67 (kindly provided by Dr. Darnell, and referred in the text as pM67) and pRL-TK (Promega, Madison, WI) were used.

### Cell Culture and Transfection

NIH3T3, NF861 and NF797 cell lines were grown in DMEM supplemented with 10% and 5% Calf Serum (Colorado Company, CO), respectively, and maintained in 10% CO_2_ humidified atmosphere. HeLa cells were grown in DMEM supplemented with 10% FCS; PC12 cells were grown in RPMI supplemented with 5% FCS and 10% Horse serum and maintained in 5% CO_2_ humidified atmosphere. Parental and derived NIH3T3 cell lines were transfected with Lipofectamine2000 (Invitrogen, Carlsbad, CA) according to manufacturer's instruction. PC12 cells were transfected with CellFECTINE reagent (Invitrogen, Carlsbad, CA). HeLa cells were transfected with FuGENE6 reagent (Roche, Manheim, Germany) according to manufacturer's instruction.

NGF was from Upstate Biotechnology; UO126 was purchased from Promega (Madison, WI); K252a and S3I-201 (also named NSC 74859) were from Calbiochem (San Diego, CA). Treatments were performed as described in Figure legends.

### Western Blot Analysis

Cell lysates were produced in RIPA modified buffer (20mM Tris-HCl, pH 7.4, 150mM NaCl, 5mM EDTA, 1% NonidetP-40) supplemented with Complete Mini EDTA-free protease Inhibitor Cocktail (Roche), 1mM NaVO4 and 1mM PMSF. Protein samples (20–50 µg) were boiled in NuPAGE LDS sample buffer (Invitrogen, Carlsbad, CA) and separated on 7%, 10% or 4–12% SDS-PAGE (NuPAGE Novex- Invitrogen, Carlsbad, CA) with the appropriate running buffer (Tris-Acetate, MOPS, MES), then transferred onto nitrocellulose filters and immunoblotted with the appropriated antibodies.

Cell fractionation was performed using the ProteoExtract Subcellular Proteome Extraction (S-PEK) Kit (Calbiochem, San Diego, CA) according to manufacturer's instructions.

The anti-STAT3, anti-phosphoSTAT3 S727, anti-phosphoSTAT3 Y705 were from Cell Signal Technology (Beverly, MA); anti-TRK (C-14), cyclinD1 (DSC-6) and anti histone H1 antibodies were purchased from Santa Cruz Biotechnology (Santa Cruz, CA); anti-vinculin, anti-phosphoERK1/2, anti ERK1/2, anti acetylated α-tubulin, anti α-tubulin, anti α-tubulin were purchased from SIGMA. The immuno-reactive bands were visualized using horseradish peroxidase-conjugated secondary antibodies and enhanced chemiluminescence (GE Healthcare).

### Luciferase Assay

Cells (5×10^4^) were seeded in 24-well plates and transfected with 150 ng of pM67 plasmid, 50 ng of pRL-TK (Promega, Madison, WI), and 250 ng of the plasmid of interest. The total amount of transfected DNA was kept constant by adding empty pCDNA3-myc vector. Forty-eight hours after transfection cell extracts were prepared and reporter gene activity was determined by Dual-Luciferase Reporter^R^ Assay (Promega, Madison, WI) according to manufacturer's protocol. The experiments were performed in triplicates. Protein expression was checked by Western blot analysis.

### RNA Extraction and RT-PCR

Total RNA was isolated with TRIZOL Reagent (Invitrogen, Carlsbad, CA), as suggested by manufacturer's protocol. One microgram of mRNA was retro-transcribed using SuperscriptIII with random examers, according to manufacturer's protocol. (Invitrogen, Carlsbad, CA). The following primers were used: AGCTACTGTAATGATCAGTCAACG (forward) and AGAGGTCCTTTTCACCAGCA (reverse) for 198 bp HPRT fragment amplification; GACCCGCCAACAAATTAAGA (forward) and TCGTGGTAAACTGGACACCA (reverse) for 215 bp STAT3 fragment amplification. After a denaturation at 94°C for 3min, 27 and 25 PCR cycles (for HPRT and STAT3 fragments respectively) were performed (94°C for 30 s, 55°C for 30 s, 72°C for 30 s), followed by a final extension at 72°C for 5 min. PCR reactions were performed in a total volume of 20 µl containing 15ng of single strand cDNAs, 1xPCR buffer, 1 mM MgCl_2_, 200 µM each dNTP, 0.5 µM each primer and 0.25U of AmpliTaq-Gold polymerase (Applied Biosystem, Foster City, CA, USA). PCR products were separated on 2% agarose gel; images were acquired by Biorad GelDoc and densitometric analysis of the bands was performed by Image Quant software.

### Immunofluorescence Assay

NF797 cells were plated on glass coverslips, treated or not with 200 nM K252a for 16 hours. Cells were fixed with 3% paraformaldehyde 2% sucrose, and stained with anti-α-tubulin monoclonal antibodies, followed by AlexaFluor546 secondary antibodies (1∶500; Molecular Probes, Eugene, OR). Coverslides were mounted with Prolong Antifade reagent with DAPI (Molecular Probes, Eugene, OR). Stained cells were observed with microscope Nikon Eclipse E1000; images were captured with Nikon DXM digital camera.

### Cell Viability Assay

To determine cell growth rate the alamarBlue®Assay (Biosource, Nivelles, Belgium) was used, following the supplier information. Four hundreds cells per well were plated in 96 wells-dishes. After adhesion cells were treated with 100 µM S3I-201 or 0.3% DMSO as control and the alamarBlue reagent was added to the medium at 20% v/v. Fluorescence at λ =  590nm and λ =  535nm were detected 6, 24 and 48 hours after treatment using a microplate reader (Tecan). Data were normalized for values detected 6 hours after addition of alamarBlue reagent.
